# Meta-analysis shows that circulating tumor cells including circulating microRNAs are useful to predict the survival of patients with gastric cancer

**DOI:** 10.1186/1471-2407-14-773

**Published:** 2014-10-21

**Authors:** Zhen-yu Zhang, Zhen-ling Dai, Xiao-wei Yin, Shu-heng Li, Shu-ping Li, Hai-yan Ge

**Affiliations:** Department of Gastrointestinal Surgery, Shanghai East Hospital, Tongji University School of Medicine, Pudong New District, No. 150, Jimo Road, Shanghai, 200120 China; Department of Research Administration, Shanghai East Hospital, Tongji University School of Medicine, Pudong New District, No. 150, Jimo Road, Shanghai, 200120 China

## Abstract

**Background:**

Circulating tumor cells (CTCs) are metastatic cells disseminated into the bloodstreams. They have been proposed to monitor disease progression for decades. However, the prognostic value of CTCs in gastric cancer (GC) remains controversial. We performed a meta-analysis to investigate the topic.

**Methods:**

A systematic search was made for relevant studies in academic data bases, involving the Medline, Embase, and Science Citation Index. Data on prognosis of GC patients, such as recurrence-free survival (RFS) and overall survival (OS), were extracted when possible. The meta-analysis was performed with the random effects model and the pooled hazard ratios (HRs) and their associated 95% confident intervals (95%CIs) were computed as effect measures.

**Results:**

Twenty six studies (including 40 subgroups) with peripheral blood samples of 1950 cases from 10 countries were included in the final analysis. The pooled results showed that GC patients with detectable CTCs (including circulating miRNAs) had a tendency to experience shortened RFS (HR = 2.91, 95% CI [1.84-4.61], I^2^ = 52.18%, n = 10). As for patient deaths, we found a similar association of CTC (including circulating miRNAs) presence with worse OS (HR = 1.78, 95% CI [1.49-2.12], I^2^ = 30.71%, n = 30). Additionally, subgroup analyses indicated strong prognostic powers of CTCs, irrespective of geographical, methodological, detection time and sample size differences of the studies.

**Conclusions:**

Our meta-analysis shows that CTCs (including circulating miRNAs) can predict the survival of GC patients. Large prospective studies are warranted to determine the best sampling time points, detection methods in homogeneous patients with GC in the future.

**Electronic supplementary material:**

The online version of this article (doi:10.1186/1471-2407-14-773) contains supplementary material, which is available to authorized users.

## Background

Gastric cancer (GC) is a very common disease with the highest rates of prevalence and mortality in East Asia [1]. Unfortunately, available routine tests including serum protein markers are not efficient enough to early detect GCs or predict metastases [2]. Most GCs are diagnosed at an advanced rather than an early stage, leading to an overall 5-year survival rate of below 30%.

Circulating tumor cells (CTCs) are metastatic cells in blood, sheltering subsets with metastasis-initiating ability [3]. They have attracted much attention not only because of their easy accessibility but also for their superiority over conventional tumor markers [4]. CellSearch system (Veridix LLC) is the only platform cleared by the FDA for CTC quantification in cancer patients. Based on the platform, CTCs have been investigated and proposed for many aspects of cancer management, such as monitoring disease recurrence [5] and therapy responses [6, 7], determining drug-selection strategies [8], and predicting the survival of cancer patients [9, 10].

Nevertheless, due to technical limitations on CTC detection, there are no widely accepted methods. Many of the major techniques, including reverse transcription-polymerase chain reaction (RT-PCR) and the CellSearch system, have been suspected of their abilities to identify CTC components with down-regulated epithelial markers generated from epithelia-mesenchyme transition (EMT). In consideration of those drawbacks, a number of studies are focused on developing antigen-independent devices (i.e., micro-infiltration and negative depletion of leukocytes) and searching for unbiased markers which are specifically enriched in CTCs. But most of them remain to be validated by clinical samples.

CTCs are also crucial contributor and indicator for GC [11]. Meanwhile, controversies still exist in the prognostic role of CTCs for GC. We recently reviewed studies on detection and clinical impact of CTCs in patients with GC [11], and found that researchers had reported diverse detection methods, tumor markers, sampling time points and results for CTCs, which were inconsistent and sometimes difficult for readers to understand. With the aim to investigate the prognostic values of CTCs and to interpret the results of available studies statistically, we performed a meta-analysis on the topic.

## Methods

### Search strategies and study selection

We made an extensive search in the Medline, Embase and Science Citation Index for studies investigating the prognostic value of CTCs in GC patients without time and language restrictions. Terms, such as “circulating tumor cells”, “blood”, “gastric cancer” and “prognosis”, were jointly searched.

To yield potential relevant publications, we screened the titles, abstracts and author information of studies we collected. Researches would not be considered for detailed assessment, unless they met the following inclusion criteria: (1) studies should investigate the prognostic significance of CTCs on GC patients with at less one outcomes (i.e., OS and RFS), (2) the forms of CTCs were tumor cells from blood mononuclear cells (MNCs), CTC-related molecular derivatives from MNCs and plasma rather than protein tumor markers in serum, and (3) studies from the same institutions were included to keep the maximum information if they reported different markers or applied different methods.

To legitimize studies for subsequent meta-analysis, we assessed the full texts and references of relevant articles (including reviews) with the following exclusion criteria: (1) duplicated publications, (2) patients enrolled were less than twenty, (3) studies on serum protein markers, (4) no survival data or insufficient data to be extracted, and (5) case reports, editorials, comments and letters were excluded.

### Data extraction

Two reviewers (Z-y Zhang and Z-l Dai) independently extracted the data. Baseline characteristics recorded for each eligible study were as follows: surname of the first author, year of publication, country of origin, number and median/mean age of patients analysed, follow-up duration, TNM stage of included subjects, detection method, markers to identify CTCs, sampling time, detection rate, endpoints and survival data. Disagreements were resolved by discussion.

### Statistical approaches

To statistically assess the prognostic effects of CTCs on the survival of GC, we extracted individual HRs and associated 95%CIs when available. Otherwise, they were estimated base on survival data or survival curves using suggested methods by Parmar [12] and Tierney et al [13]. In addition, when HRs were presented by both univariate and multivariate analyses, the latter ones were preferable because multivariate analyses also considered possible confounding of exposure effects [14].

Generally, a HR >1 indicated a worse outcome of patient with positive expression of CTCs. We pooled the extracted HRs with generic inverse variance method provided in the Comprehensive Meta-Analysis program (version 2.2, Englewood, NJ, Biostat). Potential heterogeneity across the studies was illustrated by forest plots [15]. The Cochrane’s Q statistic and I^2^ statistic were computed to test the significance [16]. The random effects model was used only when the tests were significant (two-tailed P value ≤0.1, I^2^ > 50%) [17, 18].

For studies with multiple arms (i.e., resectable and unresectable groups) or multiple markers (each marker within the study can define the positivity of CTCs), each of the subgroups was considered an independent data set. However, as for studies with multiple time points (i.e., pre-therapy and intra/post-therapy detections), we used data from pre-therapy samples in prior to intra/post-therapy samples because those data were usually dependent. To validate the priority, sensitivity analyses were conducted by alternating with data on the other time points. With regard to studies from the same institutions, sensitivity analyses by excluding all of them or only keeping the latest study were performed to make sure whether there was significant impact to destabilize the overall effects. In the present study, circulating miRNAs were treated as novel indicators of CTCs for GC. However, considering microRNAs (miRNAs) were not as specific as the other markers to indicate CTCs, we made subgroup analyses and meta-regression to assess the reliability and potential biases as well (see below).

The quality of the included studies was assessed with the Newcastle-Ottawa Scale (NOS) for cohort studies [19], which was recommended by the Cochrane Library for observational studies. To test the reliability of our results, we performed sensitivity analyses. The influences of a particular study on the summary effects were explored by calculating the combined HRs after randomly removing one included study. Sensitivity tests were also conducted by inclusion of metastatic tumors and quantified with the Duval and Tweedie’s trim and fill method [20].

Furthermore, subgroup analyses were made to explore existing heterogeneity. Studies were stratified by country of study origin, publication year, sample size, approaches, marker type, detection rate and sampling time. Subgroup analyses were performed only when there were two or more studies included in the subgroups. Univariate meta-regression analyses (random effects) on the same factors were implemented [21].

Lastly, we measured publication biases of the eligible studies using funnel plots. Biases were statistically tested by Begg’s and Egger’s methods [22]. Fail-safe numbers were calculated. We also investigated the impact of the publication year on the pooled results by cumulative meta-analyses. All of the above mentioned methods in the meta-analyses have followed the MOOSE Checklist (See Additional file 1).

## Results

### Baseline characteristics

**Figure 1:**
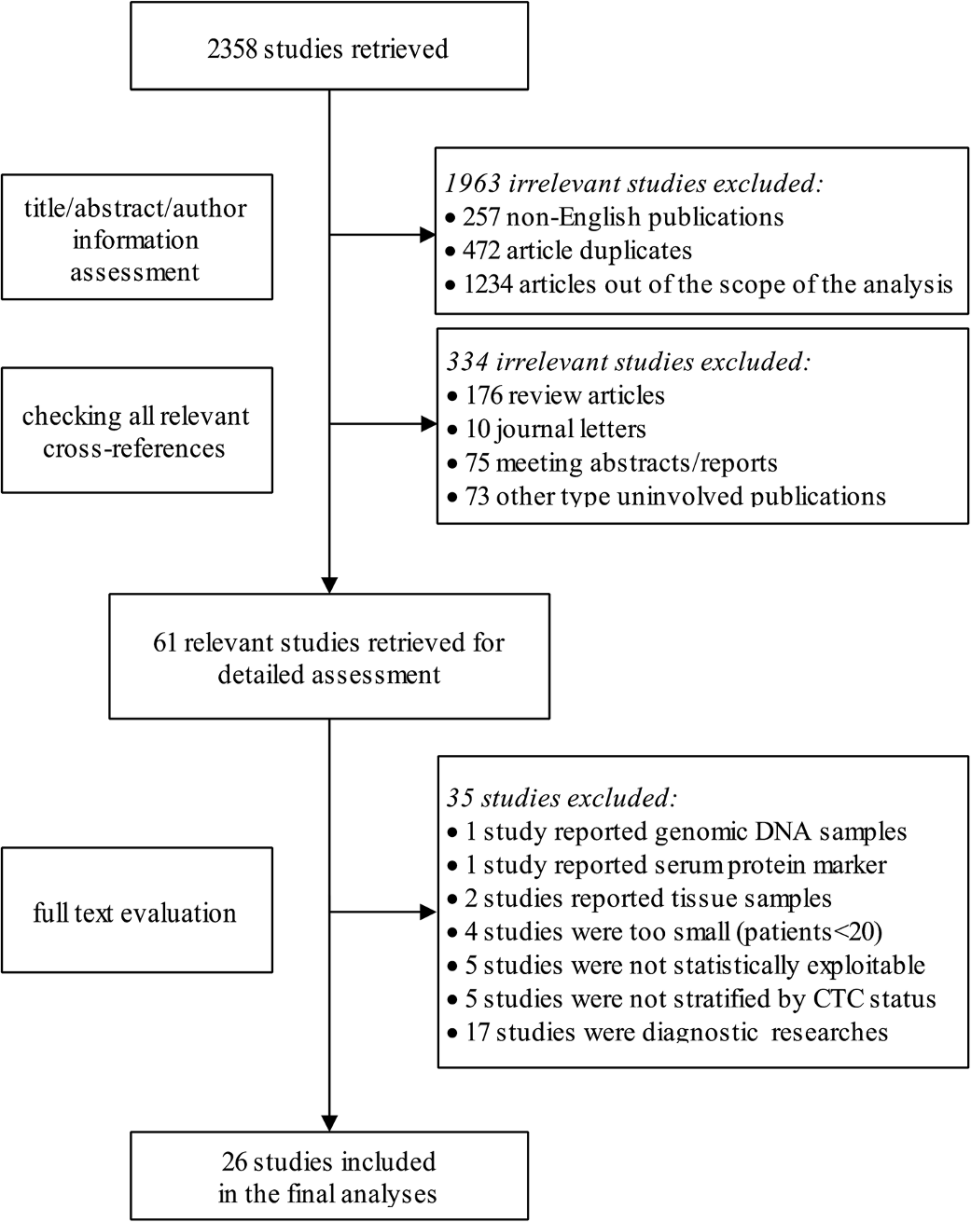
**Flowchart of study selection.**

The comprehensive search was performed on 15^th^ March 2014, yielding a total of 2538 results (See Additional files 2, 3, and 4). Among the results, 1963 studies were identified as non-English publications, duplicates and studies out of the scope of the analyses. Another 334 publications were reported as non-research articles. All of them were therefore excluded for detailed assessment. The remaining 61 reports were thoroughly assessed, of which 26 studies were legitimized into the final analyses (Figure 1).

The twenty six studies [23–48] with 1950 patients were published between the year of 2005 and 2013 in ten countries, which were located in East Asia [23, 25, 26, 28–30, 32, 34, 36, 37, 40–42, 44–48] or other areas [24, 27, 31, 33, 35, 38, 39, 43]. The median patient no. per study was 69 (range, 26 to 251). The sampling time point reported more frequently was pre-therapy [24, 27, 29, 30, 32–37, 40–46, 48] (before operations or chemotherapies, n = 18), compared to intra [25, 26, 28, 31, 34, 38, 39] or post-therapy [23, 47] (during or after operations as well as chemotherapies, n = 9). Only one study reported multiple time points including baseline, week-2 and week-4 during therapies [34]. The methods mostly used to detect CTCs were molecular techniques (n = 21), including RT-PCR [23, 24, 26, 29, 31–33, 35, 36, 38–40, 42, 44–47] methylation-specific PCR (MSP) [43], RT-PCR enzyme linked immunosorbent assay (RT-PCR ELISA) [30, 37] and high-throughput colorimetric membrane-array (HTCMA) [25]. Meanwhile, cytological means (n = 5) such as the CellSearch system [28, 34, 48], fluorescence-activated cell sorting (FACS) [27] and immunocytochemistry (ICC) [27, 41] were also reported. The commonly investigated markers were cytokeratin 18, 19, 20 (CK18/19/20), carcinoembryonic antigen (CEA) and miRNAs. Of note, unlike classic tumor markers, the expressions of miRNAs were not restricted to epithelial cells but frequently altered in malignant tumors including GC. Therefore, miRNAs were only moderately sensitive and specific for CTC detection (i.e., the sensitivity and specificity of miR-200c [39] were 65.4% and 100%, respectively). Besides, more than one markers were reported in ten researches with six [26, 27, 29, 33, 40, 41] of them defining CTC events as the positivity of any one marker. Another four studies [25, 28, 34, 42] considered CTC status to be positive only when all markers were positive. The median detection rate of CTCs irrespective of methods and time points was 50.0% (range, 10.8% to 98.6%). Of note, all eligible studies only detected CTCs in peripheral blood. In summary, nine researches reported RFS as an endpoint for GC patients while twenty two reported OS with one [45] of them presenting cancer-specific survival (CSS), which could be considered a subset of OS logically. Additionally, five studies provided both RFS and OS data. All essential characteristics of included studies (Table 1) were carefully evaluated for the following analyses.

**Table 1:** **Baseline characteristics of eligible studies**

ID [Name (Year)]	Country	Stage (UICC)	Methods	Time points	Markers	Positive rates n/N (%)	Endpoints	Hazard ratios	Quality
Ikeguchi (2005) [23]	Japan	I-IV	RT-PCR	post-therapy	CEA	25/55(45.5)	RFS/OS	data extrapolated	High
Illert (2005) [24]	Germany	I-IV	RT-PCR	Pre-therapy	CK20	15/41(36.6)	OS(R0)^a^	data extrapolated	High
				Pre-therapy	CK20	13/29(44.8)	OS(R2/UR)^a^	data extrapolated	
Wu (2006) [25]	China	I-IV	HTCMA	intra-therapy	CK19/CEA/MUC1/hTERT	39/64(60.9)	OS	data extrapolated	High
Uen (2006) [26]	China	I-IV	RT-PCR	intra-therapy	c-MET	32/52(61.5)	OS	data extrapolated	High
					MUC1	37/52(71.2)	OS	data extrapolated	
Noworolska (2007) [27]	Poland	I-IV	FACS-ICC	Pre-therapy	CK8/18/19	31/57(54.4)	OS	data extrapolated	High
Hiraiwa (2008) [28]	Japan	IV	CellSearch	intra-therapy	EpCAM/CK8/18/19	15/27(55.6)	OS	data extrapolated	Low
Koga (2008) [29]	Japan	I-IV	RT-PCR	Pre-therapy	CK19	8/69(11.6)	OS	data extrapolated	High
					CK20	10/69(15.5)	OS	data extrapolated	
Yie (2008) [30]	China	I-IV	RT-PCR	Pre-therapy	survivin	12/26(46.2)	RFS	reported in text	High
Bertazza (2009) [31]	Italy	I-IV	RT-PCR	intra-therapy	survivin	69/70(98.6)	OS	reported in text	High
Arigami (2010) [32]	Japan	I-IV	RT-PCR	Pre-therapy	B7-H4	71/94(75.5)	OS	reported in text	High
Kutun (2010) [33]	Turkey	I-IV	RT-PCR	pre-therapy	CK19	24/50(48.0)	OS	data extrapolated	Low
					CEA	10/50(20.0)	OS	data extrapolated	
Matsusaka (2010) [34]	Japan	I-IV	CellSearch	pre-therapy	EpCAM/CK8/18/19	17/52(32.7)	RFS/OS	reported in text	Low
				intra-therapy wk2^b^	EpCAM/CK8/18/19	7/51(13.7)	RFS/OS	reported in text	
				intra-therapy wk4^b^	EpCAM/CK8/18/19	9/48(18.8)	RFS/OS	reported in text	
Saad (2010) [35]	Egypt	I-IV	RT-PCR	pre-therapy	CK18	15/30(50.0)	RFS/OS	reported in text	High
Arigami (2011) [36]	Japan	I-IV	RT-PCR	pre-therapy	B7-H3	48/95(50.5)	OS	reported in text	High
Cao (2011) [37]	China	I-IV	RT-PCR	pre-therapy	survivin	45/98(45.9)	RFS	reported in text	High
Stein (2011) [38]	Germany	I-IV	RT-PCR	intra-therapy	*S100A4*	32/64(50.0)	RFS	data extrapolated	High
Ayerbes (2012) [39]	Spain	I-IV	RT-PCR	intra-therapy	miR-200c	28/52(53.8)	RFS/OS	reported in text	High
Wang (2012) [40]	China	I-IV	RT-PCR	pre-therapy	miR-20a	34/65(52.3)	OS	reported in text	High
					miR-17-5p	33/65(50.8)	OS	reported in text	
Ito (2012) [41]	Japan	I-IV	ICC	pre-therapy	telomerase	41/65(63.1)	OS	data extrapolated	High
Arigami (2013) [42]	Japan	I-IV	RT-PCR	pre-therapy	*STC2*	43/93(46.2)	OS	reported in text	High
Balgkouranidou (2013) [43]	Greece	I-IV	MSP	pre-therapy	*mSOX17*	43/73(58.9)	OS	reported in text	High
Kang (2013) [44]	China	I-IV	RT-PCR	pre-therapy	hTERT	118/118(100)	RFS/OS	reported in text	High
Komatsu (2013) [45]	Japan	I-IV	RT-PCR	pre-therapy	miR-21	47/69(68.1)	OS	reported in text	High
					miR-17-5p	38/69(55.1)	OS	data extrapolated	
					miR-106a	53/69(76.8)	OS	data extrapolated	
					miR-106b	56/69(81.2)	OS	data extrapolated	
Lee (2013) [46]	Korea	I-IV	RT-PCR	pre-therapy	*mSEPT9*	27/153(17.6)	RFS	data extrapolated	High
Song (2013) [47]	China	I-IV	RT-PCR	post-therapy	miR-21	51/103(49.5)	OS	data extrapolated	High
Uenosono (2013) [48]	Japan	I-IV	CellSearch	pre-therapy	EpCAM/CK8/18/19	16/148(10.8)	OS(R)^c^	reported in text	High
						16/148(10.8)	RFS(R)^c^	data extrapolated	
						62/103(61.8)	OS(UR)^c^	data extrapolated	

**Note**. Refer to Additional file 5: Table S1 for detailed information.

Refer to the abbreviation section for detailed abbreviations.

^a^R0 resection and R2/unresectable groups.

^b^Two weeks and four weeks after baseline.

^c^Resectable and unresectable groups.

### Overall effects

**Figure 2:**
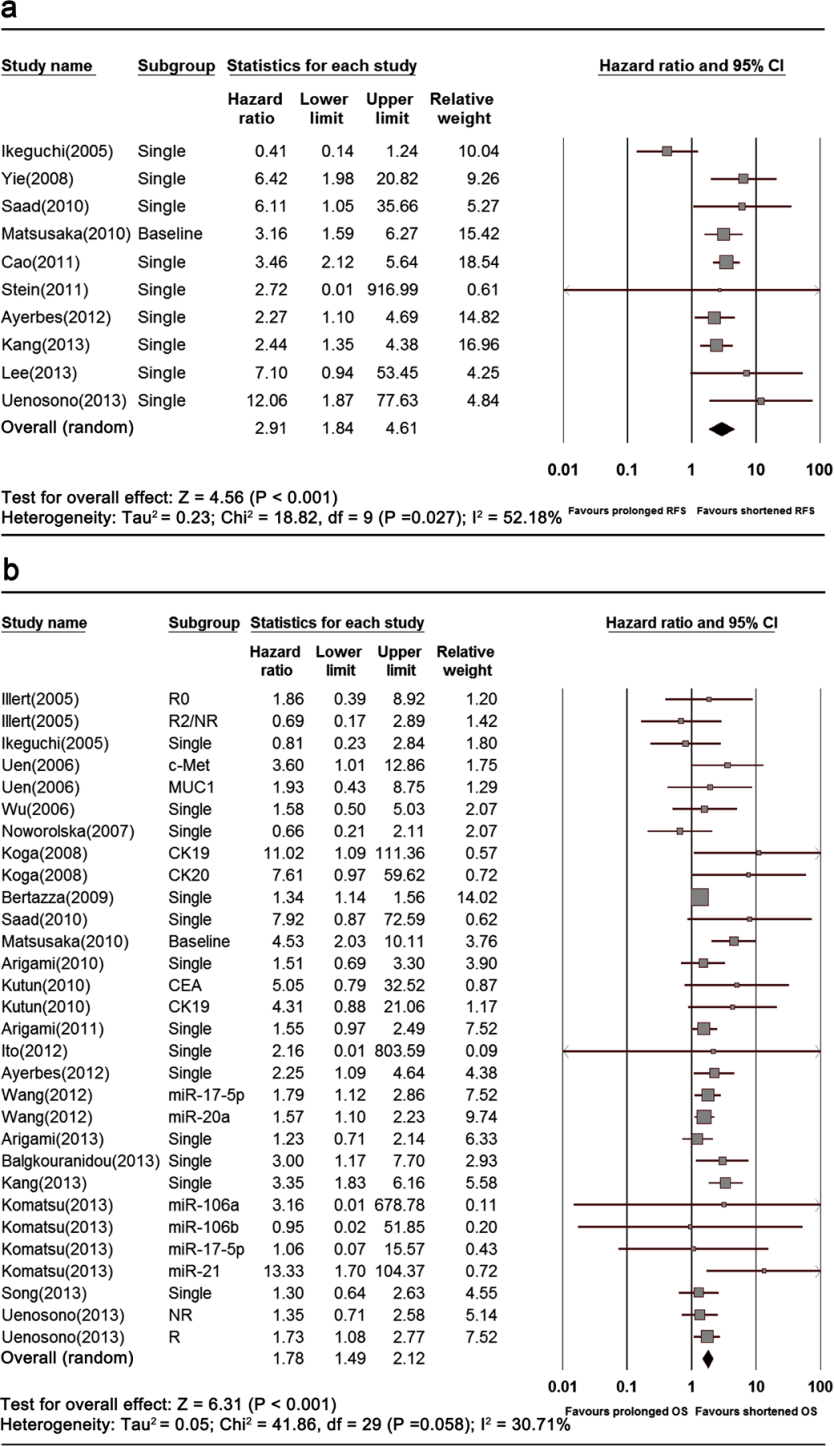
**Forest plots of RFS (a) and OS (b) in GC patients.**

The tests demonstrated heterogeneity of included studies on RFS (I^2^ = 52.18%, p = 0.027) and OS (I^2^ = 30.71%, p = 0.058), respectively. Therefore, we had to perform the meta-analyses with random effects model. The pooled results (Figure 2) showed that CTCs including circulating miRNAs were an significant prognostic factor for GC patients (RFS: HR = 2.91, 95% CI [1.84-4.61], n = 10; OS: HR = 1.78, 95% CI [1.49-2.12], n = 30).

### Subgroup analyses and meta-regression

To clarify the intra-study inconsistencies, we stratified the included studies based on variables as shown in Table 2. Heterogeneity was eliminated in subgroups by exclusion of studies published before the year of 2010, with comparable HRs but more precise CIs. Furthermore, the heterogeneity dropped to insignificant level in meta-analyses on OS when studies were stratified by country, sampling time and detection rate. Of note, when one subgroup exclusively reported East Asia patients, cytological methods, pre-therapy detection or large patient numbers (above median), the HR was more conspicuous compared with that of its paired subgroup. Furthermore, both RT-PCR and the CellSearch systems were demonstrated to be valid approaches to detect CTCs in predicting patient survival. Studies with and without miRNAs did not lead to significant changes in the overall effects although it tended to yield consistent results with the same marker type, suggesting a need for standard markers to identify CTCs in future studies. When the studies grouped by method and sampling time simultaneously (see Additional file 5: Table S3), the heterogeneity became unobvious in the RT-PCR group of PFS and OS, indicating that sampling time was an important source of inconsistency. Nevertheless, the prognostic role of CTCs for RFS was not observed in a subgroup of only three studies [23, 38, 39] without pre-therapy samples (HR = 1.09, 95%CI[0.25-4.73], I^2^ = 69.09%). This might because that the follow-up period for study by Ikeguchi et al. was very short (less than 24 months) and the censored rate in the study by Stein et al. was relatively high with significant loss of patient information.

**Table 2:** **Results of subgroup analyses on RFS and OS**

Variables	RFS				OS			
	HR[95%CI]	n	I ^2^	P ^d^	HR[95%CI]	n	I ^2^	P ^d^
**Year > median** ^**a**^								
No	2.45[1.13-5.31]	4	78.87%	0.003	1.82[1.34-2.46]	15	45.79%	0.027
Yes	3.02[2.18-4.18]	6	0.00%	0.519	1.72[1.46-2.04]	15	0.00%	0.479
**Country**								
East Asia	2.93[1.67-5.14]	7	66.13%	0.007	1.76[1.50-2.07]	21	18.48%	0.220
Non-East Asia	2.62[1.35-5.10]	3	0.00%	0.596	1.41[1.22-1.63]	9	39.70%	0.103
**Methodology**								
Cytological	3.71[1.95-7.06]	2	42.92%	0.186	1.77[1.16-2.70]	5	54.00%	0.069
Molecular	2.64[1.52-4.59]	8	57.03%	0.023	1.54[1.37-1.72]	25	26.23%	0.114
**Approach**								
RT-PCR	2.61[1.52-4.59]	8	57.04%	0.023	1.79[1.46-2.20]	24	29.30%	0.090
CellSearch	3.71[1.95-7.01]	2	42.92%	0.186	2.00[1.28-3.13]	3	64.90%	0.058
Others	/	/	/	/	1.04[0.46-2.34]	3	0.00%	0.564
**Marker type**								
Non-miRNA	3.08[1.80-5.26]	9	56.48%	0.019	1.80[1.45-2.25]	22	41.33%	0.023
miRNA	/	/	/	/	1.70[1.33-2.16]	8	0.00%	0.602
**Time point**								
Pre-therapy	3.45[2.54-4.67]	7	0.00%	0.529	1.81[1.54-2.13]	23	28.58%	0.100
Intra/post-therapy	1.09[0.25-4.73]	3	69.09%	0.039	1.38[1.19-1.60]	7	0.00%	0.539
**Patient no. > median** ^**b**^								
No	2.40[1.24-4.65]	5	71.90%	0.007	1.73[1.44-2.08]	18	9.70%	0.339
Yes	3.24[2.26-4.66]	5	0.00%	0.482	1.82[1.37-2.42]	12	47.91%	0.032
**Detection rate > median** ^**c**^								
No	2.69[1.33-5.44]	5	74.38%	0.004	1.75[1.42-2.15]	14	33.73%	0.105
Yes	2.82[1.87-4.27]	5	0.00%	0.524	1.49[1.31-1.70]	16	27.31%	0.149
**Overall**	2.91[1.84-4.61]	10	52.18%	0.027	1.78[1.49-2.12]	30	30.71%	0.058

^a^The median year for both RFS and OS was 2010.

^b^The median patient no. per study for RFS and OS was 59 and 65, respectively.

^c^The median detection rate for RFS and OS was 46.05% and 51.55%, respectively.

^d^Two-tailed P value of tests for heterogeneity.

The results of subgroup analyses were in accordance with the meta-regression to quantify heterogeneity across studies (Table 3). As for studies on RFS, only time point of blood collection was significantly correlated with intra-study variability (slope = 0.9260, P = 0.007). While, the country of origin (slope = 0.2241, P = 0.004), time point (slope = 0.2733, P = 0.014) and positive rate of CTCs (slope = -0.0102, P = 0.010) contributed to heterogeneity across studies on OS. Besides, it seemed that inclusion of less specific miRNAs did not contribute to significant heterogeneity by meta-regression on RFS (p = 0.507) and OS (p = 0.444) studies.

**Table 3:** **Results of meta-regression on RFS and OS**

Variables	RFS			OS		
	Slope	SE ^a^	P value	Slope	SE ^a^	P value
Year	0.1650	0.0854	0.053	0.0377	0.0340	0.267
Country	−0.0101	0.5767	0.986	0.2241	0.1113	0.004
Method	−0.4894	0.5604	0.382	−0.0180	0.2176	0.934
Marker type	−0.2650	0.3991	0.507	0.1061	0.1386	0.444
Time point	0.9260	0.3442	0.007	0.2733	0.1115	0.014
Patient no.	0.0045	0.0061	0.465	−0.0011	0.0031	0.726
Detection rate	−0.0206	0.0155	0.185	−0.0102	0.0020	0.010

^a^Standard error of the slope.

### Quality assessment and sensitivity analyses

To test whether the results were stable with known heterogeneity, we performed sensitivity analyses. Three researches [28, 33, 34] were identified as low quality reports (NOS score ≤4, see Additional file 5: Table S2). Sensitivity analysis by excluding these low quality studies showed that the pooled effects were stable (RFS: HR = 2.92, 95% CI [1.69-5.04], I^2^ = 57.27%, n = 9; OS: HR = 1.51, 95% CI [1.36-1.69], I^2^ = 18.03%, n = 27). When we evaluating the impact of including studies from same institutions [25, 26, 32, 36, 42, 48] as stated above, the results only changed slightly by retaining the latest reports [25, 42] (RFS: HR = 2.71, 95% CI [1.72-4.27], I^2^ = 51.42%, n = 9; OS: HR = 1.88, 95% CI [1.48-2.40], I^2^ = 42.07%, n = 24). Further subgroup analyses and meta-regression did not reach any significance in institution. Removing all of the 6 studies did not contribute to significant changes of the pooled measures (OS: the same institution, HR = 1.55, 95% CI[1.23-1.96], n = 8, different institution, HR = 1.93, 95% CI[1.53-2.44], n = 22; RFS, different institution, HR = 2.71, 95% CI [1.72-4.27], n = 9) although there was a tendency that the 6 studies from the same populations were likely to present homogeneous results (OS, same vs. different, I^2^ = 0.00% & P = 0.901 vs. I^2^ = 46.20% & P = 0.010), indicating that future studies could benefit from recruitment of homogeneous populations.

Moreover, conversions of statistical method to a fixed effects model did not change the overall effects obviously (RFS: HR = 2.85, 95% CI [2.17-3.74]; OS: HR = 1.56, 95%CI [1.40-1.74]). Inclusion of the study [28] with single stage IV subjects only yielded a very close result (OS: HR = 1.78, 95% CI [1.49-2.12], I^2^ = 28.76%, p = 0.058). The other analyses with removing one study (see Additional file 6: Figures S1 and S2) and trim and fill method (see Additional file 6: Figures S3 and S4) also indicated that our results were stable.

### Publication biases

Publication biases existed in OS group, as indicated by Begg’s rank correlations (RFS: P = 0.721; OS: p = 0.193) and Egger’s regression tests (RFS: P = 0.664; OS: p = 0.007). We thus computed the fail-safe numbers for both (RFS: n = 115; OS: n = 462). The calculations showed that only when a minimum of 462 studies with negative results were included, would the overall effects on OS be negative. Moreover, the year of publication (see Additional file 6: Figures S5 and S6) did not led to any publication bias according to cumulative meta-analyses.

## Discussion

Our current meta-analysis provides strong evidence that CTCs including circulating miRNAs in peripheral blood are significantly associated with adverse RFS and OS of GC patients, irrespective of the geographical, methodological, detection time and sample size differences.

In theory, CTCs take numerous advantages to be distinctive markers in translational researches. But in practice, the history of CTCs remains elusive and the detection of CTCs still faces technical challenges. Previous investigations on breast and gastrointestinal cancers have been meta-analysed [49–52] to elaborate some practical problems. Here, we are focused on the prognostic significance of CTCs in GC patients. Compared to another two meta-analyses on the similar topic of GC [51, 52], we have applied many advanced statistical methods in the present meta-analysis, such as “one study removed”, trim and fill method, meta-regression, fail-safe numbers as well as cumulative meta-analyses. These methods are helpful to get deeper and more comprehensive insights into the prognostic value of CTCs and potential heterogeneity of included studies.

There are some novel findings in our meta-analysis. CTCs have shown significant utilities to prognose survival, but it needs further clarification that which experimental factors should be adjusted for accurate estimations of survival benefits. Thus, we conducted subgroup analyses by publication year, country, country, patient size, detection rate and marker type in addition to detection method and time point. We found more pronounced HRs in some studies, which exclusively reported East Asia patients, cytological methods, pre-therapy CTC detection and large study population. We further observed that studies tended to be consistent if they were published after 2010, with pre-therapy detections or higher detection rates. The subgroup analyses also indicated considerable intra-study heterogeneity caused by differences of geography, sampling time and detection rate from included studies. Through meta-regression, we finally confirmed and quantified the extent of sampling time (0.926 for RFS and 0.2733 for OS, respectively) which had positively contributed to heterogeneity.

However, methodological differences were not significant in both subgroup analyses and meta-regression. Similar results were obtained from another meta-regression which reported CTCs in breast cancer [50]. One possible reason may be that both methods are antigen-dependent, which enables them to detect some CTC subsets with prognostic meanings. Nevertheless, the known tumor markers used to identify CTCs also bring about a degree of bias, for they are unable to recognize CTC subsets with down-regulated markers (i.e., EMT cells). Besides, available approaches to CTC detection have been questioned for their low sensitivity and yields. Of note, our data suggested that cytological identification of CTCs seemed to be superior to molecular methods. It may be reasonable because morphological examinations are more conserved while it is easier for molecular techniques to give rise to false positive results from non-neoplastic and contaminated samples. In spite of the heterogeneity from CTCs phenotypes and methodology, employment of standardized methods should be helpful to lower intra-study inconsistencies.

Importantly, we observed remarkable heterogeneity from time points of blood collection in groups of OS, with more prominent HRs from pre-therapy detection. The CTC detection rates of pre-therapy (RFS/OS: median = 45.90/50.80%, mean = 33.60/49.20%) tended to be lower than those of intra/post-therapy (RFS/OS: median = 45.50/53.8%, mean = 37.41/54.30%) based on our included studies. It is believed that surgeries contribute to elevated CTC detection rates shortly afterwards [53], and have long-term effects on reduction of CTC burden and promotion of survival in operable subjects. But it should be noted that such promotion by surgical manipulations tends to be associated with increased detectable levels of CTC molecular derivatives, as is proved by a mouse model [54]. Since molecular methods (i.e., RT-PCR) are unable to recognize viable and functional CTCs, the detection of CTCs immediately after surgeries may provide very limited information to predict pathologic consequences (i.e., distant metastases and deaths caused by cancer) with this method. For instance, Ikeguchi et al. [23] observed transient positive conversions of CTCs status shortly after GC surgeries. But based on data collected shortly after surgeries, the authors found that survival of patients with detectable CTCs was better than those without CTCs, which had further led to wrong conclusions. Therefore, it is at least improper to detect CTCs soon after surgeries. Of cause, the differences in CTCs positive rates in different time points are mainly because of the long term anti-tumor treatments. CTCs can be eliminated by chemotherapeutic drugs through direct and indirect mechanisms, such as cytotoxic and antimetabolic effects. Surgeries by excision of primary and metastatic tumors directly stop the releasing of CTCs and cut off the bilateral communications between CTCs and tumor masses. However, if cancers fail to be cured, CTCs may increase to high levels as a result of tumor progression or recovery of tumor cells from dormancy. In theory, CTC tests before interventions contain baseline information of CTC burden. Their presence at this time point actually indicates ongoing or already established blood borne metastases, which usually cannot be effectively controlled or thoroughly eliminated. Since metastasis contributes to most cancer deaths, it may be more pathologically meaningful to characterize CTCs prior to any treatments. Consequently, time point of blood collection should be an important factor for researchers to estimate patient survival. But post-therapy monitoring of CTCs at proper time points is also very important because constantly increasing CTC burden probably indicates tumor recurrences, which will worsen patient survival if left untreated. Some authors have concerned that baseline detection have risks of failing to provide information about the actual burden of CTCs after therapies thus might be unable to accurately predict survival of patients post treatments [49, 55]. As few reports have investigated multiple time points and most natural history of CTCs remains elusive, the controversies on better time points for CTC detection have not been well understood biologically and pathologically. Further studies are needed to expound whether there are significant differences among different time points within the same patients and whether patients can benefit from such differences.

We also noticed inconsistencies from the countries of included patients in OS group. We pooled studies from different populations, which usually resulted in non-ignorable errors on total effects. But in our subgroup analyses, the prognostic role of CTCs remained significant regardless of regional differences. In addition, heterogeneity was observed from CTC detection rates. To a certain extent, inconsistent detection rates may in turn reflect heterogeneous populations, detection methods and time points. As a result, large prospective studies are expected to compare the impact of such differences on survival in homogeneous GC patients.

It should be pointed out that there are some limitations of our meta-analysis that allow us to interpret the results with caution. We used data extracted from heterogeneous studies, where individual patient data were usually not available. The total number of patients from retrievable data was relatively small. Large prospective studies were absent for GC. Besides, there were only 10 eligible studies in the meta-analysis on RFS, of which the results were limited. Although there was no standardized tool to assess the quality of non-randomized and observational studies, the sensitivity analyses demonstrated that the results were stable. To control biases generated by study retrieval and data extraction, we had developed extensive search strategies in advance to yield as much information as possible by independent reviewers. We only included studies with over 20 patients. To avoid data dredging, we had presetted limited variables before meta-regression. Although publication biases appeared in the study group of OS, the estimation of fail-safe number confirmed no obvious influences on our results.

## Conclusions

In conclusion, our meta-analysis has evidenced the significant prognostic power of CTCs including circulating miRNAs for both RFS and OS in GC patients. Large prospective studies are needed to validate the prognostic values of CTCs with multiple time points in homogeneous GC patients. But above all, bias-controlled markers and standardized detection platforms are expected to normalize and reduce the inconsistencies across studies.

## Electronic supplementary material

Additional file 1: **Meta-analysis of Observational Studies in Epidemiology (MOOSE) Checklist.** (DOC 56 KB)

Additional file 2: **Search strategies and results of Embase.** (DOC 42 KB)

Additional file 3: **Search strategies and results of Medline.** (DOC 42 KB)

Additional file 4: **Search strategies and results of Science Citation Index.** (DOC 39 KB)

Additional file 5: Table S1: Variables of included subgroups. **Table S2.** Quality assessment of included cohort studies with the Newcastle-Ottawa Scale (NOS). **Table S3.** Subgroup analyses by approaches and time points. (DOC 164 KB)

Additional file 6: Figure S1: Sensitivity analysis on RFS by randomly removing one study. **Figure S2.** Sensitivity analysis on OS by randomly removing one study. **Figure S3.** Funnel plot of RFS with observed and imputed studies. Black solid circulars refer to studies imputed for a symmetrical funnel plot. **Figure S4.** Funnel plot of OS with observed and imputed studies. Black solid circulars refer to studies imputed for a symmetrical funnel plot. **Figure S5.** Cumulative meta-analysis of OS by publication year. **Figure S6.** Cumulative meta-analysis of RFS by publication year. (DOC 2 MB)

## Abbreviations

B7-H3: CD276
B7-H4: VTCN1
CEA: Carcinoembryonic antigen
CI: Confident interval
CK8: Cytokeratin 8
CK18: Cytokeratin 18
CK19: Cytokeratin 19
c-MET: MNNG HOS Transforming gene
CSS: Cancer-specific survival
CTCs: Circulating tumor cells
EMT: Epithelia-mesenchyme transition
EpCAM: Epithelial cell adhesion molecule
FACS: Fluorescence-activated cell sorting
GC: Gastric cancer
HR: Hazard ratio
HTCMA: High-throughput colorimetric membrane-array
hTERT: Human telomerase reverse transcriptase
ICC: Immunocytochemistry
miR-17-5p: microRNA-17-5p
miR-20a: microRNA-20a
miR-21: microRNA-21
miR-106a: microRNA-106a
miR-106b: microRNA-106b
miR-200c: microRNA-200c
MNCs: Mononuclear cells
mSEPT9: Methylated septin-9
mSOX17: Methylated SRY(sex determining region Y)-box 17
MSP: Methylation-specific PCR
MUC1: Mucin 1
OS: Overall survival
RFS: Recurrence-free survival
RT-PCR: Reverse transcription-polymerase chain reaction
RT-PCR ELISA: RT-PCR enzyme linked immunosorbent assay
S100A4: S100 calcium binding protein A4
STC2: Stanniocalcin-2
Survivin: BIRC5.
