# Supplementary material for: Meta-analysis shows that circulating tumor cells including circulating microRNAs are useful to predict the survival of patients with gastric cancer
Source: BMC Cancer. 2014 Oct 21;14:773. doi: 10.1186/1471-2407-14-773 (PMC4210594; doi:10.1186/1471-2407-14-773)
Supplement: Supplementary file 1 — Additional file 1: Meta-analysis of Observational Studies in Epidemiology (MOOSE) Checklist. (DOC 56 KB) [file 12885_2014_4947_MOESM1_ESM.doc]

### Additional file 1 –Meta-analysis of Observational Studies in Epidemiology (MOOSE) Checklist

**Manuscript title: Meta-analysis shows that circulating tumor cells including circulating microRNAs are useful to predict the survival of patients with gastric cancer**

| **Criteria** | | **Brief description of how the criteria were handled in the meta-analysis** |
| --- | --- | --- |
| **Reporting of background should include** | |  |
| √ | Problem definition | Circulating tumor cells (CTCs) have been considered contributors and indicators for cancer metastasis. However, the prognostic role of CTCs remains controversial in gastric cancer patients. |
| √ | Hypothesis statement | We hypothesize that CTCs can predict the survival of patients with gastric cancer. |
| √ | Description of study outcomes | Any prognostic outcomes that can be treated as overall survival (OS), recurrence-free survival (RFS). |
| √ | Type of exposure or intervention used | Patients at exposure refer to those with detectable CTCs or altered levels of molecular derivatives (including miRNAs but no protein markers), which are identified by any kind of cytological and molecular methods. |
| √ | Type of study designs used | No limitations on study designs except non-research articles such as letters, case reports and comments et al. |
| √ | Study population | No restrictions. |
| **Reporting of search strategy should include** | |  |
| √ | Qualifications of searchers | Librarian and information specialist. The credentials of the three investigators (who contributed to the search strategy) are indicated in the author list. |
| √ | Search strategy, including time period included in the synthesis and keywords | Embase Classic+Embase from 1947 to 2014 March 14  Medline from 1945 to 2014 March 7  Science Citaion Index^TM^ Core Collection from 1986 to 2014 March 12  See Additional file 2-4 |
| √ | Databases and registries searched | Embase Classic+Embase, Medline and Science Citaion Index^TM^ Core Collection |
| √ | Search software used, name and version, including special features | OvidSP and Thomson Reuters Web of Knowledge platform. We detailed the MeSH/ Emtree Headings and text words/key words in Additional file 2-4 |
| √ | Use of hand searching | References of the retrieved papers were hand searched for additional studies. |
| √ | List of citations located and those excluded, including justifications | Details of the literature search process are outlined in the flow chart (Figure 1). The citation list of excluded articles is available upon request. |
| √ | Method of addressing articles published in languages other than English | We set no search restrictions on language and we obtained all included articles in English language. |
| √ | Method of handling abstracts and unpublished studies | We did not include unpublished or abstract only studies. |
| √ | Description of any contact with authors | We contacted the original authors for clarification when it was needed. |
| **Reporting of methods should include** | |  |
| √ | Description of relevance or appropriateness of studies assembled for assessing the hypothesis to be tested | Detailed inclusion and exclusion criteria have been described in the manuscript. |
| √ | Rationale for the selection and coding of data | Data extracted from each eligible study were any essential clinical factors, characteristics and survival data, which were relevant to the survival of gastric cancer patients. See table 2 and table S1. |
| √ | Assessment of confounding | We mainly conducted subgroup analyses and meta-regression to evaluate the confounding factors. See table 2, table 3, table S3 and figure S1-S4. |
| √ | Assessment of study quality, including blinding of quality assessors; stratification or regression on possible predictors of study results | We used the Newcastle Ottawa Scale (NOS) to assess the quality of each study. We conducted sensitivity analysis by removing the studies in low quality. See table S2. |
| √ | Assessment of heterogeneity | We applied the Q statistic and I^2^ value to assess the heterogeneity. |
| √ | Description of statistical methods in sufficient detail to be replicated | We explained detailed methods and the software we used to process the data in the manuscript. |
| √ | Provision of appropriate tables and graphics | We included one flow chart to show the method of studies selection, figure 2 to show the forest plots, table 1 table to show the baseline characteristics of included studies, table 2 to show the results of subgroup analyses, table 3 to show the results of meta-regression and addition files 1-6 to show the results of study search and sensitivity analysis et al. |
| **Reporting of results should include** | |  |
| √ | Graph summarizing individual study estimates and overall estimate | Figure 2 |
| √ | Table giving descriptive information for each study included | Table 1 and Table S1 |
| √ | Results of sensitivity testing | These were described in the manuscript and figure S1-S4 |
| √ | Indication of statistical uncertainty of findings | 95% CI intervals were presented for all meta-analyses together with I^2^ values. |
| **Reporting of discussion should include** | |  |
| √ | Quantitative assessment of bias | Subgroup and meta-regression were performed. Risks of publication biases were also assessed. |
| √ | Justification for exclusion | Reasons for exclusion were shown in the flow chart of study selection, with the two major reasons. One was studies only reported diagnostic data. Another on was that no or inadequate data could be extracted. |
| √ | Assessment of quality of included studies | See above mentioned parts. |
| **Reporting of conclusions should include** | |  |
| √ | Consideration of alternative explanations for observed results | We discussed in the manuscripts that ununiformed patients, multiple time points, different methods, and markers might contribute to bias of included studies. |
| √ | Generalization of the conclusions | Our results evidenced the prognostic role of CTCs in gastric cancer. |
| √ | Guidelines for future research | Large prospective studies are needed to validate the prognostic values of CTCs with multiple time points in homogeneous GC patients. But above all, bias-controlled markers and standardized detection platforms are expected to normalize and reduce the inconsistencies across studies. |
| √ | Disclosure of funding source | Funding sources were stated in the manuscript. |
