# Supplementary material for: Meta-analysis shows that circulating tumor cells including circulating microRNAs are useful to predict the survival of patients with gastric cancer
Source: BMC Cancer. 2014 Oct 21;14:773. doi: 10.1186/1471-2407-14-773 (PMC4210594; doi:10.1186/1471-2407-14-773)
Supplement: Supplementary file 2 — Additional file 2: Search strategies and results of Embase. (DOC 42 KB) [file 12885_2014_4947_MOESM2_ESM.doc]

### Additional file 2 –Search strategies and results of Embase

1. Data base: **Embase Classic+Embase** (via OVIDSP platform)
2. Time span: < 1947 to 2014 March 14 >

Searches were performed on 2014-03-15 at the Library of Tongji University School of Medicine, Shanghai

**Search strategies and results**

| **Set** | **Searches** | **Results** |
| --- | --- | --- |
| 1 | minimal residual disease.mp. [mp=title, abstract, subject headings, heading word, drug trade name, original title, device manufacturer, drug manufacturer, device trade name, keyword] | 13729 |
| 2 | occult disease.mp. [mp=title, abstract, subject headings, heading word, drug trade name, original title, device manufacturer, drug manufacturer, device trade name, keyword] | 542 |
| 3 | ((Blood or hemato* or heamato*) adj3 (tumo* cell* or cancer* cell* or carcinom* cell* or neoplas* cell*)).mp. [mp=title, abstract, subject headings, heading word, drug trade name, original title, device manufacturer, drug manufacturer, device trade name, keyword] | 15304 |
| 4 | (Shedd* adj3 cell*).mp. [mp=title, abstract, subject headings, heading word, drug trade name, original title, device manufacturer, drug manufacturer, device trade name, keyword] | 1461 |
| 5 | ((Circulat* or isolated or disseminat* or occult or metastatic) adj3 (tumo* cell* or cancer* cell* or carcinom* cell* or neoplas* cell* or mRNA* or microRNA* or DNA*)).mp. [mp=title, abstract, subject headings, heading word, drug trade name, original title, device manufacturer, drug manufacturer, device trade name, keyword] | 38167 |
| 6 | 1 OR 2 OR 3 OR 4 OR 5 | 66130 |
| 7 | (Blood* or hemato* or heamato* or circulat*).mp. [mp=title, abstract, subject headings, heading word, drug trade name, original title, device manufacturer, drug manufacturer, device trade name, keyword] | 4330363 |
| 8 | 7 AND 6 | 33591 |
| 9 | ((Gastr* or digesti* or stomach*) adj3 (tumo* or cancer* or carcinom* or neoplas*)).mp. [mp=title, abstract, subject headings, heading word, drug trade name, original title, device manufacturer, drug manufacturer, device trade name, keyword] | 225457 |
| 10 | exp gastric cancer/ | 74834 |
| 11 | 10 OR 9 | 227693 |
| 12 | 8 AND 11 | 1505 |
| 13 | (survival* or prognos* or recurren*).mp. [mp=title, abstract, subject headings, heading word, drug trade name, original title, device manufacturer, drug manufacturer, device trade name, keyword] | 1926152 |
| 14 | ((predict* or risk* or clinic*) adj3 (factor* or marker* or biomarker* or value* or role* or significan*)).mp. [mp=title, abstract, subject headings, heading word, drug trade name, original title, device manufacturer, drug manufacturer, device trade name, keyword] | 1279767 |
| 15 | exp prognosis/ | 465185 |
| 16 | 13 OR 14 OR 15 | 2959972 |
| 17 | 12 AND 16 | 765 |
| 18 | ((Gastr* or digesti* or stomach*) adj3 (tumo* or cancer* or carcinom* or neoplas*)).ti. | 66655 |
| 19 | 17 AND 18 | 208 |
| 20 | (mouse or mice or rat* or animal*).ti. | 1699481 |
| 21 | 19 NOT 20 | 205 |

**Note.** mp: the term should appears in title, abstract, subject headings, heading word, drug trade name, original title, device manufacturer, drug manufacturer, device trade name, or keyword of the manuscript.

adj3: position operator, the term should be adjacent to the other with less than three words between them.

*: right-hand truncation

exp: explode

/: mesh term

ti: search terms in the title of the manuscript.
