# Supplementary material for: Meta-analysis shows that circulating tumor cells including circulating microRNAs are useful to predict the survival of patients with gastric cancer
Source: BMC Cancer. 2014 Oct 21;14:773. doi: 10.1186/1471-2407-14-773 (PMC4210594; doi:10.1186/1471-2407-14-773)
Supplement: Supplementary file 3 — Additional file 3: Search strategies and results of Medline. (DOC 42 KB) [file 12885_2014_4947_MOESM3_ESM.doc]

### Additional file 3 –Search strategies and results of Medline

1. Data Base: **Medline** (via Thomson Reuters Web of Knowledge platform)
2. Time span: < 1945 to 2014 March 7 >

The Searches were performed on 2014-03-15 at the Library of Tongji University School of Medicine, Shanghai

**Search strategies and results**

| **Set** | **Results** | **Search History** |
| --- | --- | --- |
| #1 | 6489 | MH=(Neoplasm, Residual) |
| #2 | 9103 | TS=Occult disease |
| #3 | 186,033 | TS=((Blood OR hemato* OR heamato*) SAME (tumo* cell* OR cancer* cell* OR carcinom* cell* OR neoplas* cell*)) |
| #4 | 8,125 | TS=(Shedd* SAME cell*) |
| #5 | 352,841 | TS=((Circulat* OR isolated OR disseminat* OR occult OR metastatic) SAME (tumo* cell* OR cancer* cell* OR carcinom* cell* OR neoplas* cell* OR mRNA* OR microRNA* OR DNA* )) |
| #6 | 6,327 | MH=(Neoplastic Cells, Circulating) |
| #7 | 520,089 | #1 OR #2 OR #3 OR #4 OR #5 OR #6 |
| #8 | 3,300,361 | TS=(Blood* OR hemato* OR heamato* OR circulat*) |
| #9 | 237,482 | #7 AND #8 |
| #10 | 179,686 | TS=((Gastr* OR digesti* OR stomach*) SAME (tumo* OR cancer* OR carcinom* OR neoplas*)) |
| #11 | 71,472 | MH:exp=(Stomach Neoplasms) |
| #12 | 179,686 | #11 OR #10 |
| #13 | 10,347 | #12 AND #9 |
| #14 | 1,488,728 | TS=(survival* OR prognos* OR recurren*) |
| #15 | 2,697,041 | TS=((predict* OR risk* OR clinic*) SAME (factor* OR marker* OR biomarker* OR value* OR role* OR significan*)) |
| #16 | 126,868 | MH=(Neoplasm Recurrence, Local OR blood) |
| #17 | 1,028,128 | MH:exp=(Prognosis) |
| #18 | 4,084,700 | #17 OR #16 OR #15 OR #14 |
| #19 | 4,651 | #18 AND #13 |
| #20 | 55,856 | TI=((Gastr* OR digesti* OR stomach*) SAME (tumo* OR cancer* OR carcinom* OR neoplas*)) |
| #21 | 1,320 | #20 AND #19 |
| #22 | 3,835,684 | MH:exp=(Animals) NOT MH=(Humans) |
| #23 | 1,304 | #21 NOT #22 |

**Note.** MH: mesh term

TS: topic search in Title, Vernacular Title, Abstract, Other Abstract, MeSH Terms, Keyword List, Chemical, Gene Symbol, Personal Name Subject and Space Flight Mission.

SAME: the terms should appear in the same sentence.

TI: search terms in the title of the manuscript.

:exp: to explode mesh term.
