# Supplementary material for: Meta-analysis shows that circulating tumor cells including circulating microRNAs are useful to predict the survival of patients with gastric cancer
Source: BMC Cancer. 2014 Oct 21;14:773. doi: 10.1186/1471-2407-14-773 (PMC4210594; doi:10.1186/1471-2407-14-773)
Supplement: Supplementary file 4 — Additional file 4: Search strategies and results of Science Citation Index. (DOC 39 KB) [file 12885_2014_4947_MOESM4_ESM.doc]

### Additional file 4 –Search strategies and results of Science Citation Index

1. Data Base: **Science Citaion Index^TM^ Core Collection** ( via Thomson Reuters Web of Knowledge platform)
2. Time span: < 1986 to 2014 March 12 >

Searches were performed on 3014-03-15 at the Library of Tongji University School of Medicine, Shanghai

**Search strategies and results**

| **Set** | **Results** | **Search History** |
| --- | --- | --- |
| #1 | 7,718 | TS=minimal residual disease |
| #2 | 5,795 | TS=occult disease |
| #3 | 115,859 | TS=((Blood OR hemato* OR heamato*) SAME (tumo* cell* OR cancer* cell* OR carcinom* cell* OR neoplas* cell*)) |
| #4 | 7,160 | TS=(Shedd* SAME cell*) |
| #5 | 254,441 | TS=((Circulat* OR isolated OR disseminat* OR occult OR metastatic) SAME (tumo* cell* OR cancer* cell* OR carcinom* cell* OR neoplas* cell* OR mRNA* OR microRNA* OR DNA* )) |
| #6 | 360,553 | #1 OR #2 OR #3 OR #4 OR #5 |
| #7 | 1,377,646 | TS=(Blood* OR hemato* OR heamato* OR circulat*) |
| #8 | 155,023 | #7 AND #6 |
| #9 | 114,180 | TS=((Gastr* OR digesti* OR stomach*) SAME (tumo* OR cancer* OR carcinom* OR neoplas*)) |
| #10 | 6,940 | #9 AND #8 |
| #11 | 1,045,320 | TS=(survival* OR prognos* OR recurren*) |
| #12 | 2,218,589 | TS=((predict* OR risk* OR clinic*) SAME (factor* OR marker* OR biomarker* OR value* OR role* OR significan*)) |
| #13 | 2,924,019 | #12 OR #11 |
| #14 | 3,504 | #13 AND #10 |
| #15 | 38,446 | TI=((Gastr* OR digesti* OR stomach*) SAME (tumo* OR cancer* OR carcinom* OR neoplas*)) |
| #16 | 872 | #15 AND #14 |
| #17 | 1,098,810 | TI=(mouse OR mice OR rat* OR animal*) |
| #18 | 849 | #16 NOT #17 |
