# Supplementary material for: Meta-analysis shows that circulating tumor cells including circulating microRNAs are useful to predict the survival of patients with gastric cancer
Source: BMC Cancer. 2014 Oct 21;14:773. doi: 10.1186/1471-2407-14-773 (PMC4210594; doi:10.1186/1471-2407-14-773)
Supplement: Supplementary file 5 — Additional file 5: Table S1: Variables of included subgroups. Table S2. Quality assessment of included cohort studies with the Newcastle-Ottawa Scale (NOS). Table S3. Subgroup analyses by approaches and time points. (DOC 164 KB) [file 12885_2014_4947_MOESM5_ESM.doc]

### Additional file 5

**Table S1 -** Variables of included subgroups

| **Study ID** | **Country** | **Age (year)** | **Follow-up, month** | **Tumor stage** | **Detection Method** | **Blood volume** | **Cut-off** | **Sampling site** | **Sampling time** | **Marker** | **Positive rate, n/N(%)** | **Endpoints** | **Hazard ratio** | **Curative surgery** | **Multivariate** | **Refs.** |
| --- | --- | --- | --- | --- | --- | --- | --- | --- | --- | --- | --- | --- | --- | --- | --- | --- |
| **Name(Year)** |  | **Mean /Median (range)** | **Mean/Median(range)** |  |  |  |  |  |  |  |  |  |  |  |  |  |
| Ikeguchi(2005) | Japan | 66/NR(26-86) | 20/NR(2-31) | I-IV | RT-PCR | 1.5 mL | / | PB | post-OP | CEA | 25/55(45.5) | RFS | data extrapolated | yes | no | [23] |
| Ikeguchi(2005) | Japan | 66/NR(26-86) | 20/NR(2-31) | I-IV | RT-PCR | 1.5 mL | / | PB | post-OP | CEA | 25/55(45.5) | OS | data extrapolated | yes | no | [23] |
| Illert(2005) | Germany | NR/69(41-87) | NR/20(1-57) | I-IV | RT-PCR | 9 mL | / | PB | pre-OP | CK20 | 15/41(36.6) | OS(R0) | data extrapolated | yes | no | [24] |
| Illert(2005) | Germany | NR/69(41-87) | NR/20(1-57) | I-IV | RT-PCR | 9 mL | / | PB | pre-treatment | CK20 | 13/29(44.8) | OS(R2/NR) | data extrapolated | yes | no | [24] |
| Wu(2006) | China | 60/NR(36-84) | NR/28(20-33) | I-IV | HTCMA | 4 mL | 5CTCs/1mL | PB | intra-OP | CK19/CEA  /MUC1/hTERT | 39/64(60.9) | OS | data extrapolated | no | no | [25] |
| Uen(2006) | China | 60/NR(34-84) | NR(>24) | I-IV | RT-PCR | 4 mL | / | PB | intra-OP | c-MET | 32/52(61.5) | OS | data extrapolated | no | no | [26] |
| Uen(2006) | China | 60/NR(34-84) | NR(>24) | I-IV | RT-PCR | 4 mL | / | PB | intra-OP | MUC1 | 37/52(71.2) | OS | data extrapolated | no | no | [26] |
| Noworolska(2007) | Poland | NR | NR(>24) | I-IV | FACS-ICC | NR | 3 CTCs /slide | PB | pre-OP | CK8/18/19 | 31/57(54.4) | OS | data extrapolated | no | no | [27] |
| Hiraiwa(2008) | Japan | NR | NR(<24) | IV | CellSearch | 7.5 mL | 2 CTCs /7.5mL | PB | pre-OP/chem/post-chem | EpCAM/CK8/18/19 | 15/27(55.6) | OS | data extrapolated | no | yes | [28] |
| Koga(2008) | Japan | 66/NR | NR(>24) | I-IV | qRT-PCR | 10 mL | / | PB | pre-OP | CK19 | 8/69(11.6) | OS | data extrapolated | yes | no | [29] |
| Koga(2008) | Japan | 66/NR | NR(>24) | I-IV | qRT-PCR | 10 mL | / | PB | pre-OP | CK20 | 10/69(15.5) | OS | data extrapolated | yes | no | [29] |
| Yie(2008) | China | NR/58(26-77) | NR(>24) | I-IV | RT-PCR ELISA | 2 mL | / | PB | pre-treatment | survivin | 12/26(46.2) | RFS | reported in text | no | yes | [30] |
| Bertazza(2009) | Italy | NR/68(28-90) | NR/15(6-119) | I-IV | qrtPCR | 6 mL | / | PB | intra-OP | survivin | 69/70(98.6) | OS | reported in text | no | yes | [31] |
| Arigami(2010) | Japan | 68/NR(35-87) | NR/25(1-74) | I-IV | qRT-PCR | 5 mL | / | PB | pre-OP | B7-H4 | 71/94(75.5) | OS | reported in text | yes | yes | [32] |
| Kutun(2010) | Turkey | 61/NR(42-84) | NR(>24) | I-IV | RT-PCR | NR | / | PB | pre-OP | CK19 | 24/50(48.0) | OS | data extrapolated | no | no | [33] |
| Kutun(2010) | Turkey | 61/NR(42-84) | NR(>24) | I-IV | RT-PCR | NR | / | PB | pre-OP | CEA | 10/50(20.0) | OS | data extrapolated | no | no | [33] |
| Matsusaka(2010) | Japan | NR/62(24-78) | NR(<24) | I-IV | CellSearch | 10 mL | 4 CTCs/7.5mL | PB | Baseline pre-chem | EpCAM/CK8/18/19 | 17/52(32.7) | RFS | reported in text | no | no | [34] |
| Matsusaka(2010) | Japan | NR/62(24-78) | NR(<24) | I-IV | CellSearch | 10 mL | 4 CTCs/7.5mL | PB | Baseline pre-chem | EpCAM/CK8/18/19 | 17/52(32.7) | OS | reported in text | no | no | [34] |
| Matsusaka(2010) | Japan | NR/62(24-78) | NR(<24) | I-IV | CellSearch | 10 mL | 4 CTCs/7.5mL | PB | Week-2 post-chem | EpCAM/CK8/18/19 | 7/51(13.7) | RFS | reported in text | no | yes | [34] |
| Matsusaka(2010) | Japan | NR/62(24-78) | NR(<24) | I-IV | CellSearch | 10 mL | 4 CTCs/7.5mL | PB | Week-2 post-chem | EpCAM/CK8/18/19 | 7/51(13.7) | OS | reported in text | no | yes | [34] |
| Matsusaka(2010) | Japan | NR/62(24-78) | NR(<24) | I-IV | CellSearch | 10 mL | 4 CTCs/7.5mL | PB | Week-4 post-chem | EpCAM/CK8/18/19 | 9/48(18.8) | RFS | reported in text | no | yes | [34] |
| Matsusaka(2010) | Japan | NR/62(24-78) | NR(<24) | I-IV | CellSearch | 10 mL | 4 CTCs/7.5mL | PB | Week-4 post-chem | EpCAM/CK8/18/19 | 9/48(18.8) | OS | reported in text | no | yes | [34] |
| Saad(2010) | Egypt | 55/NR(31-72) | NR(>24) | I-IV | RQ-PCR | 2 mL | / | PB | pre-chem | CK18 | 15/30(50.0) | RFS | reported in text | yes | yes | [35] |
| Saad(2010) | Egypt | 55/NR(31-72) | NR(>24) | I-IV | RQ-PCR | 2 mL | / | PB | pre-chem | CK18 | 15/30(50.0) | OS | reported in text | yes | yes | [35] |
| Arigami(2011) | Japan | 68/NR(35-67) | NR/24(1-74) | I-IV | qRT-PCR | 5 mL | / | PB | pre-OP | B7-H3 | 48/95(50.5) | OS | reported in text | yes | yes | [36] |
| Cao(2011) | China | NR | 47.5/NR(36.5-56) | I-IV | RT-PCR ELISA | 6 mL | / | PB | pre-OP | survivin | 45/98(45.9) | RFS | reported in text | yes | yes | [37] |
| Stein(2011) | Germany | NR/63(40-86) | NR(>24) | I-IV | qrtPCR | 5 mL | / | PB | intra-OP | cf-S100A4 mRNA | 32/64(50.0) | RFS | data extrapolated | yes | no | [38] |
| Ayerbes(2012) | Spain | 65/NR(49-74) | 26/NR(6-53) | I-IV | qRT-PCR | 10 mL | / | PB | post-OP/pre-chem | cf-miR-200c | 28/52(53.8) | RFS | reported in text | no | yes | [39] |
| Ayerbes(2012) | Spain | 65/NR(49-74) | 26/NR(6-53) | I-IV | qRT-PCR | 10 mL | / | PB | post-OP/pre-chem | cf-miR-200c | 28/52(53.8) | OS | reported in text | no | yes | [39] |
| Wang(2012) | China | NR | NR(>24) | I-IV | rtPCR | 2 mL | / | PB | pre-OP | cf-miR-20a | 34/65(52.3) | OS | reported in text | no | yes | [40] |
| Wang(2012) | China | NR | NR(>24) | I-IV | rtPCR | 2 mL | / | PB | pre-OP | cf-miR-17-5p | 33/65(50.8) | OS | reported in text | no | no | [40] |
| Ito(2012) | Japan | 59/NR(33-76) | NR(<24) | I-IV | ICC | 7.5 mL | 5 CTCs/1mL | PB | pre-OP | telomerase | 41/65(63.1) | OS | data extrapolated | no | no | [41] |
| Arigami(2013) | Japan | 68/NR(35-87) | NR/25(1-74) | I-IV | qRT-PCR | 5 mL | / | PB | pre-OP | STC2 | 43/93(46.2) | OS | reported in text | yes | yes | [42] |
| Balgkouranidou(2013) | Greece | 67/70(28-82) | NR/56(20-111) | I-IV | MSP | NR | / | PB | pre-OP | cf-SOX17 | 43/73(58.9) | OS | reported in text | yes | yes | [43] |
| Kang(2013) | China | NR | NR/24(4-60) | I-IV | qRT-PCR | 6 mL | / | PB | pre-OP | cf-hTERT | 118/118(100) | RFS | reported in text | yes | yes | [44] |
| Kang(2013) | China | NR | NR/24(4-60) | I-IV | qRT-PCR | 6 mL | / | PB | pre-OP | cf-hTERT | 118/118(100) | OS | reported in text | yes | yes | [44] |
| Komatsu(2013) | Japan | NR | NR(>24) | I-IV | qRT-PCR | 7 mL | / | PB | pre-OP | miR-21 | 47/69(68.1) | OS | reported in text | no | yes | [45] |
| Komatsu(2013) | Japan | NR | NR(>24) | I-IV | qRT-PCR | 7 mL | / | PB | pre-OP | miR-17-5p | 38/69(55.1) | OS | data extrapolated | no | no | [45] |
| Komatsu(2013) | Japan | NR | NR(>24) | I-IV | qRT-PCR | 7 mL | / | PB | pre-OP | miR-106a | 53/69(76.8) | OS | data extrapolated | no | no | [45] |
| Komatsu(2013) | Japan | NR | NR(>24) | I-IV | qRT-PCR | 7 mL | / | PB | pre-OP | miR-106b | 56/69(81.2) | OS | data extrapolated | no | no | [45] |
| Lee(2013) | Korea | 61/NR | 14/NR(13-15) | I-IV | rtPCR | 9 mL | / | PB | pre-therapy | mSEPT9 | 27/153(17.6) | RFS | data extrapolated | yes | no | [46] |
| Song(2013) | China | 60/NR(27-87) | NR/36(24-53) | I-IV | qrtPCR | 5 mL | / | PB | post-OP | cf-miR-21 | 51/103(49.5) | OS | data extrapolated | no | no | [47] |
| Uenosono(2013) | Japan | 64/NR(28-87) | NR/32(4-72) | I-IV | CellSearch | 7.5 mL | 1 CTC/7.5mL | PB | pre-chem | EpCAM/CK8/18/19 | 16/148(10.8) | OS(R) | reported in text | yes | yes | [48] |
| Uenosono(2013) | Japan | 64/NR(28-87) | NR/32(4-72) | I-IV | CellSearch | 7.5 mL | 1 CTC/7.5mL | PB | pre-chem | EpCAM/CK8/18/19 | 16/148(10.8) | RFS(R) | data extrapolated | yes | no | [48] |
| Uenosono(2013) | Japan | 64/NR(28-87) | NR/32(4-72) | I-IV | CellSearch | 7.5 mL | 1 CTC/7.5mL | PB | pre-chem | EpCAM/CK8/18/19 | 62/103(61.8) | OS(NR) | data extrapolated | no | no | [48] |

**Note.** NR, not reported.

qRT-PCR, quantitative RT-PCR.

qrtPCR, quantitative real-time PCR.

RQ-PCR, real time quantitative PCR.

rtPCR, real-time PCR

PB, peripheral blood

pre-OP, pre-operation

pre-chem, pre-chemotherapy

**Table S2 - Quality assessment of included cohort studies with the** Newcastle-Ottawa Scale (NOS)

| **Studies** | **Score for Selection** | | | | **Score for Comparability** | | **Score for Outcome** | | | **Aggregate score** | **Quality** |
| --- | --- | --- | --- | --- | --- | --- | --- | --- | --- | --- | --- |
|  | **Item 1** | **Item 2** | **Item 3** | **Item 4** | **Item 1** | **Item 2** | **Item 1** | **Item 2** | **Item 3** |  |  |
| Ikeguchi(2005)[23] | 0 | 1 | 1 | 1 | 0 | 0 | 1 | 0 | 1 | 5 | High |
| Illert(2005)[24] | 1 | 1 | 1 | 1 | 0 | 0 | 1 | 1 | 1 | 7 | High |
| Wu(2006)[25] | 1 | 1 | 1 | 1 | 0 | 0 | 0 | 0 | 1 | 5 | High |
| Uen(2006)[26] | 1 | 1 | 1 | 1 | 0 | 0 | 1 | 0 | 1 | 6 | High |
| Noworolska(2007)[27] | 1 | 1 | 1 | 1 | 0 | 0 | 1 | 1 | 1 | 7 | High |
| Hiraiwa(2008)[28] | 0 | 1 | 1 | 1 | 0 | 0 | 1 | 0 | 0 | 4 | Low |
| Koga(2008)[29] | 1 | 1 | 1 | 1 | 0 | 0 | 0 | 1 | 1 | 6 | High |
| Yie(2008)[30] | 1 | 1 | 1 | 1 | 0 | 0 | 0 | 1 | 0 | 5 | High |
| Bertazza(2009)[31] | 0 | 1 | 1 | 1 | 0 | 0 | 0 | 1 | 0 | 5 | High |
| Arigami(2010)[32] | 1 | 1 | 1 | 1 | 0 | 0 | 0 | 1 | 0 | 5 | High |
| Kutun(2010)[33] | 1 | 1 | 1 | 1 | 0 | 0 | 0 | 0 | 0 | 4 | Low |
| Matsusaka(2010)[34] | 0 | 1 | 1 | 1 | 0 | 0 | 0 | 0 | 1 | 4 | Low |
| Saad(2010)[35] | 1 | 1 | 1 | 1 | 0 | 0 | 1 | 0 | 1 | 6 | High |
| Arigami(2011)[36] | 1 | 1 | 1 | 1 | 0 | 0 | 1 | 1 | 0 | 6 | High |
| Cao(2011)[37] | 1 | 1 | 1 | 1 | 0 | 0 | 0 | 1 | 0 | 5 | High |
| Stein(2011)[38] | 1 | 1 | 1 | 1 | 0 | 0 | 1 | 1 | 0 | 6 | High |
| Ayerbes(2012)[39] | 1 | 1 | 1 | 1 | 0 | 0 | 1 | 1 | 1 | 7 | High |
| Wang(2012)[40] | 1 | 1 | 1 | 1 | 0 | 0 | 0 | 1 | 0 | 5 | High |
| Ito(2012)[41] | 1 | 1 | 1 | 1 | 0 | 0 | 1 | 0 | 0 | 5 | High |
| Arigami(2013)[42] | 1 | 1 | 1 | 1 | 0 | 0 | 1 | 1 | 0 | 6 | High |
| Balgkouranidou(2013)[43] | 0 | 1 | 1 | 1 | 0 | 0 | 1 | 1 | 0 | 5 | High |
| Kang(2013)[44] | 1 | 1 | 1 | 1 | 0 | 0 | 1 | 1 | 1 | 7 | High |
| Komatsu(2013)[45] | 1 | 1 | 1 | 1 | 0 | 0 | 0 | 1 | 0 | 5 | High |
| Lee(2013)[46] | 1 | 1 | 1 | 1 | 0 | 0 | 1 | 0 | 0 | 5 | High |
| Song(2013)[47] | 1 | 1 | 1 | 1 | 0 | 0 | 0 | 1 | 0 | 5 | High |
| Uenosono(2013)[48] | 1 | 1 | 1 | 1 | 0 | 0 | 1 | 1 | 0 | 6 | High |

**Note.** Numbered items in each category of the NOS are listed below.

**Selection**

Item 1) Representativeness of the exposed cohort

Item 2) Selection of the non-exposed cohort

Item 3) Ascertainment of exposure

Item 4) Demonstration that outcome of interest was not present at start of study

**Comparability**

Comparability of cohorts on the basis of the design or analysis

Item 1) study controls for the most important factor (i.e., age)

Item 2) study controls for any additional factor (treatments for cancer)

**Outcome**

Item 1) Assessment of outcome

Item 2) Was follow-up long enough for outcomes to occur (maximum follow-up period was over 36 month)

Item 3) Adequacy of follow up of cohorts (over 90%)

**Reference**

Wells G, Shea B, O’connell D, Peterson J, Welch V, Losos M, Tugwell P: **The Newcastle-Ottawa Scale (NOS) for assessing the quality of nonrandomised studies in meta-analyses**. In*.*; 2000.

**Table S3 -Subgroup analyses by approaches and time points**

| **Approaches** | **RFS (HR[95%CI])** | | **OS (HR[95%CI])** | |
| --- | --- | --- | --- | --- |
|  | **Pre-therapy** | **Intra/post-therapy** | **Pre-therapy** | **Intra/post-therapy** |
| RT-PCR | 3.37[2.39-4.77]  n=5, I^2^=0.00%, P=0.509 | 1.20[0.53-2.73]  n=3, I^2^=69.09%, P=0.039 | 1.83[1.52-2.21]  n=18, I^2^=23.00%, P=0.182 | 1.38[1.19-1.60]  n=6, I^2^=0.00%, P=0.418 |
| CellSearch | 3.71[1.95-7.01]  n=2, I^2^=42.91%, P=0.186 | / | 2.08[1.13-3.84]  n=3, I^2^=64.90%, P=0.058 | / |
| Others | / | / | 0.69[0.223-2.156]  n=2, I^2^=0.00%, P=0.701 | / |
