# Supplementary material for: Meta-analysis shows that circulating tumor cells including circulating microRNAs are useful to predict the survival of patients with gastric cancer
Source: BMC Cancer. 2014 Oct 21;14:773. doi: 10.1186/1471-2407-14-773 (PMC4210594; doi:10.1186/1471-2407-14-773)
Supplement: Supplementary file 6 — Additional file 6: Figure S1: Sensitivity analysis on RFS by randomly removing one study. Figure S2. Sensitivity analysis on OS by randomly removing one study. Figure S3. Funnel plot of RFS with observed and imputed studies. Black solid circulars refer to studies imputed for a symmetrical funnel plot. Figure S4. Funnel plot of OS with observed and imputed studies. Black solid circulars refer to studies imputed for a symmetrical funnel plot. Figure S5. Cumulative meta-analysis of OS by publication year. Figure S6. Cumulative meta-analysis of RFS by publication year. (DOC 2 MB) [file 12885_2014_4947_MOESM6_ESM.doc]

**Additional file 6**

**
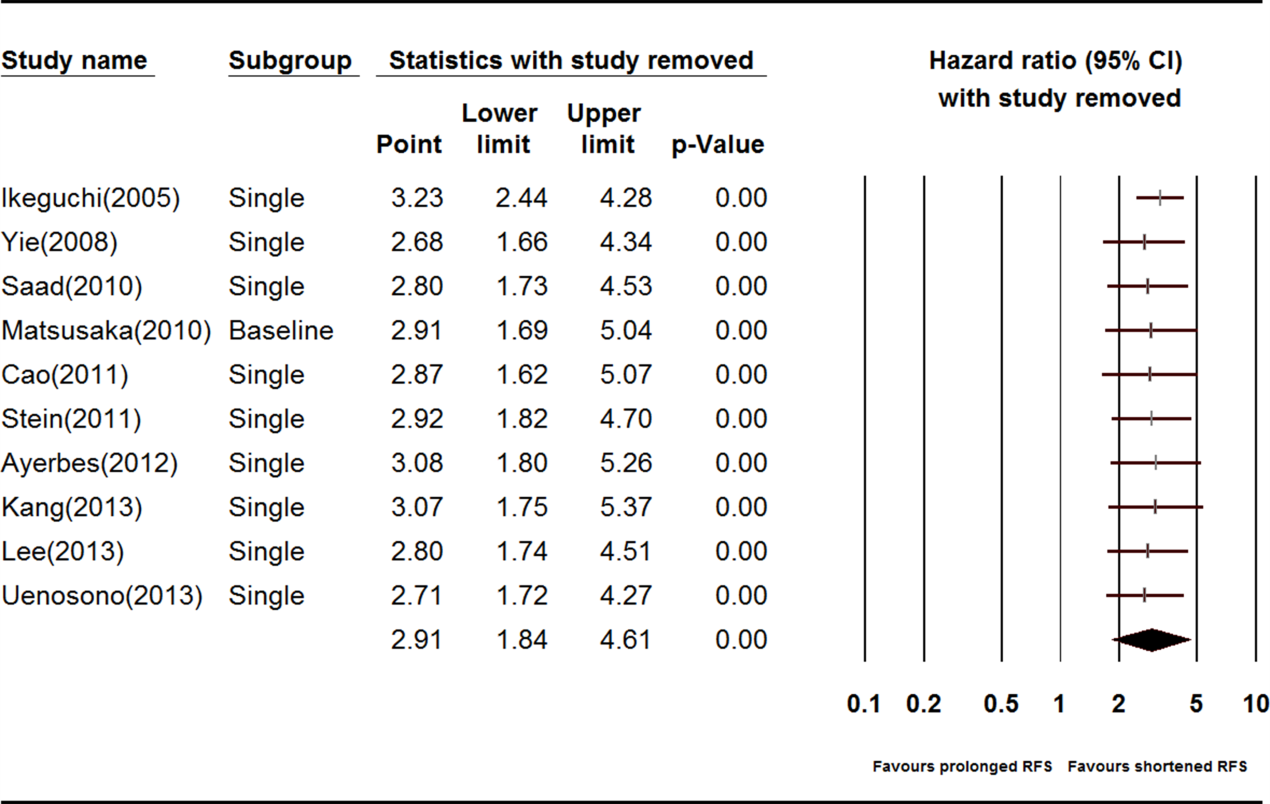
**

**Figure S1 - Sensitivity analysis on RFS by randomly removing one study**


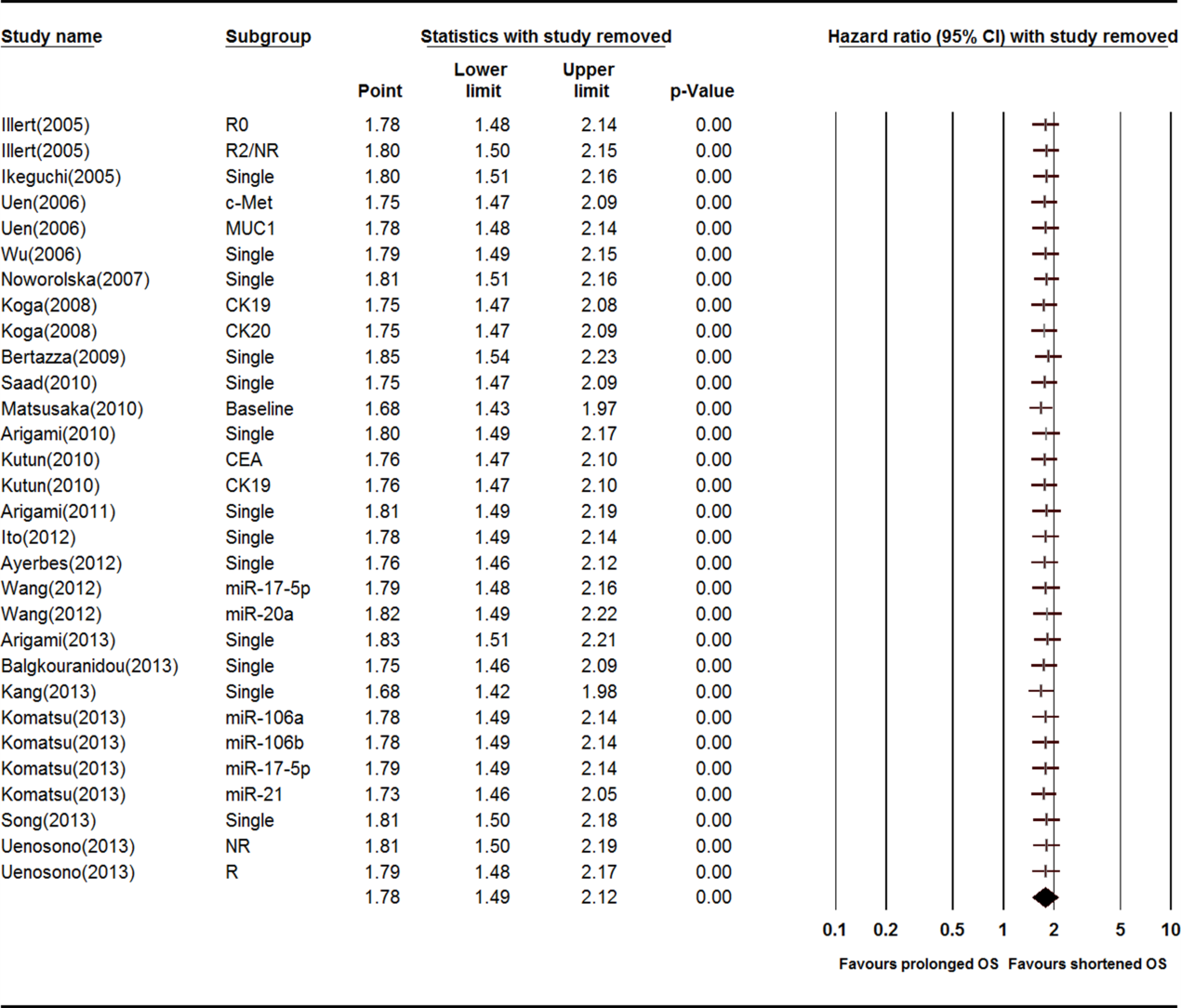


**Figure S2 - Sensitivity analysis on OS by randomly removing one study**


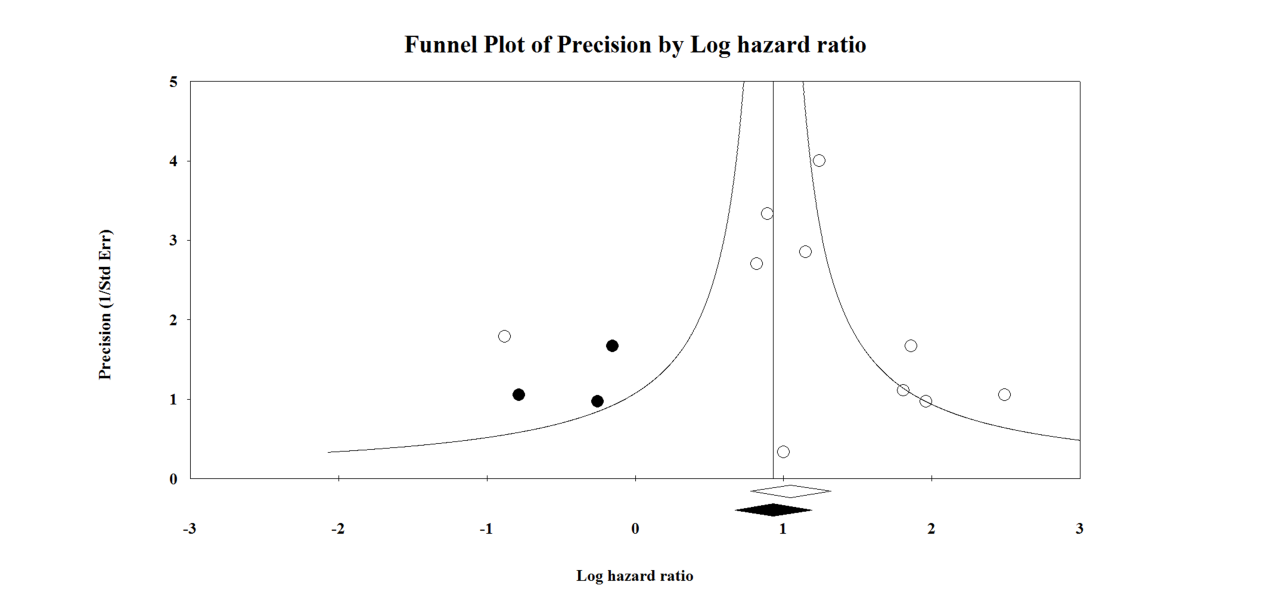


**Figure S3 - Funnel plot of RFS with observed and imputed studies**

Black solid circulars refer to studies imputed for a symmetrical funnel plot.


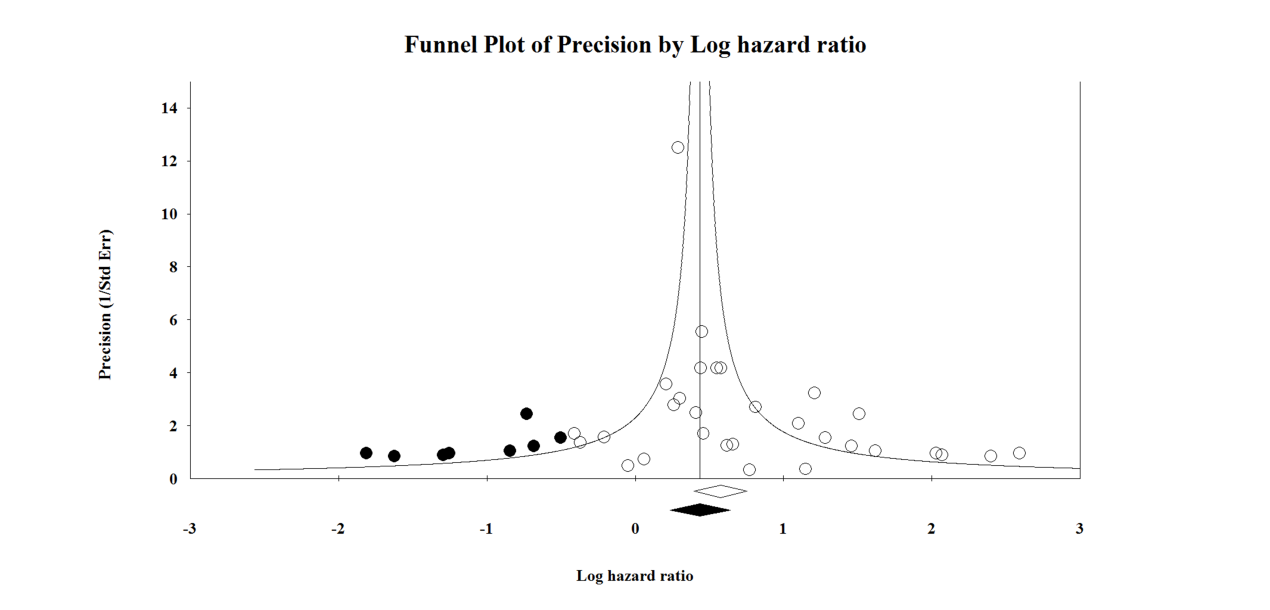


**Figure S4 - Funnel plot of OS with observed and imputed studies**

Black solid circulars refer to studies imputed for a symmetrical funnel plot.


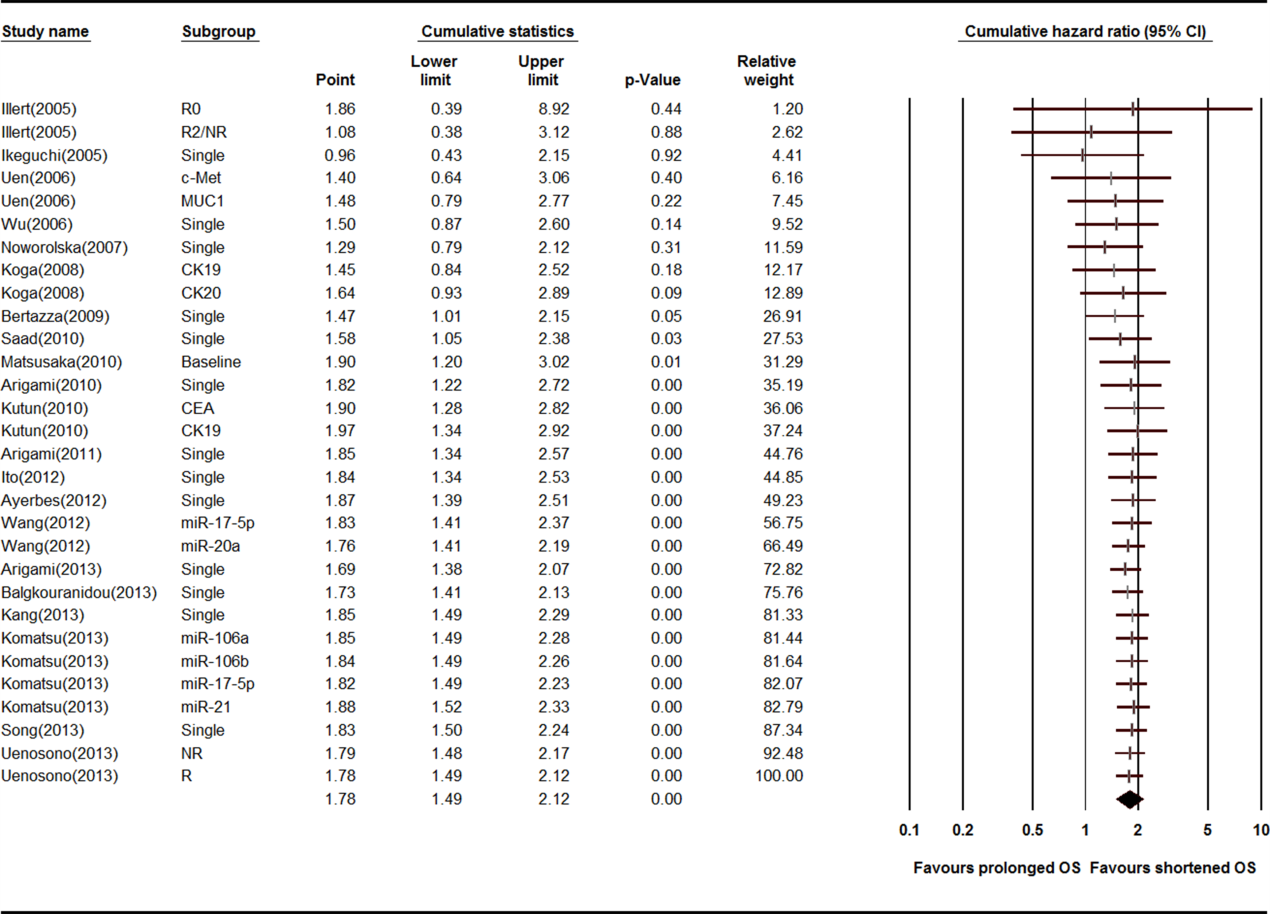


**Figure S5 - Cumulative meta-analysis of OS by publication year**


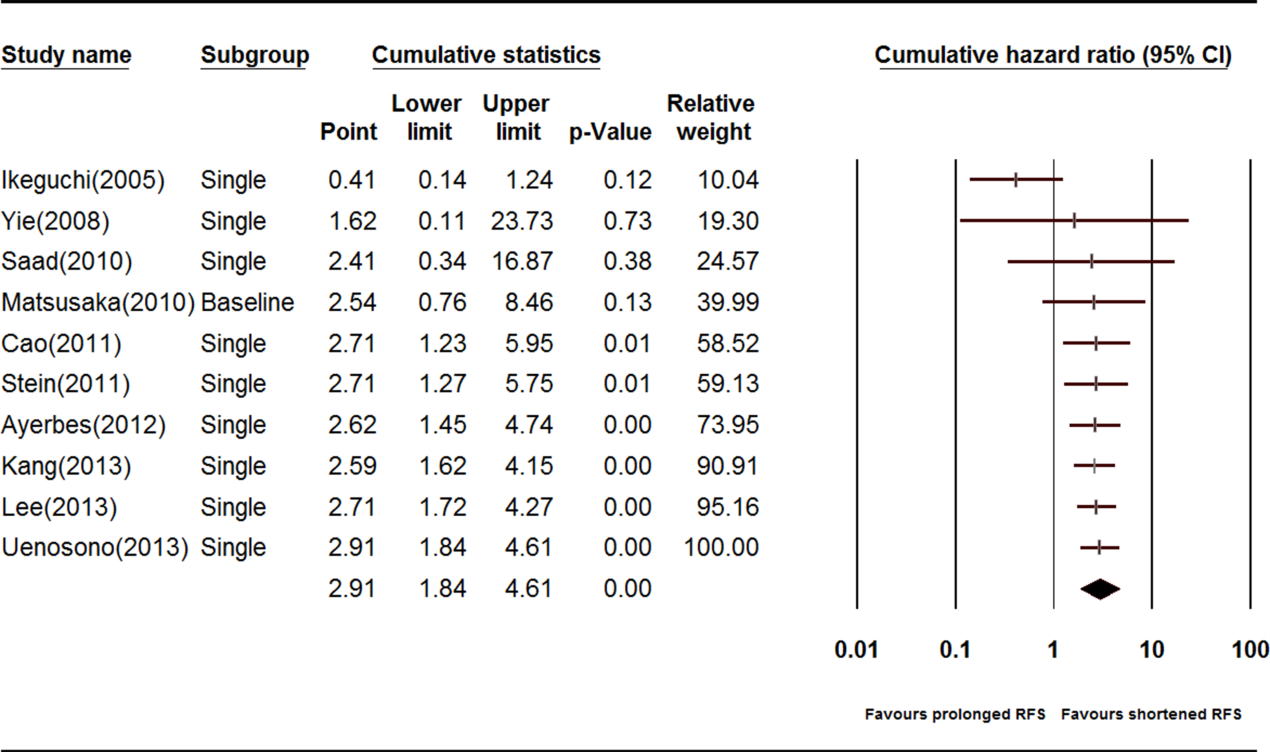


**Figure S6 - Cumulative meta-analysis of RFS by publication year**
